# A New Sub-Department of Medicine

**Published:** 1911-10-14

**Authors:** 


### A NEW SUB-DEPARTMENT OF MEDICINE.

From its foundation in the seventies of the last century right up to the present day the Johns Hopkins University has always been one of the most progressive educational centres in the United States, and its medical branch has done much towards the earning of this reputation. Moreover, in the effort towards constant improvement there has never been a relapse into mere faddism. A character, a solidity, and a scholarly strain pervade the work of the Johns Hopkins, such as are conspicuously deficient in some of the other American universities. The latest illustration of this excellent trait is the announcement of a new department created there. An Associate-Professor has been appointed to take charge of the subject "Art as applied to Medicine"; and the first occupant of the professorial chair is the well-known Max Brödel. The purpose of the new professor is to bridge over the gap existing between art and medicine, to train a new generation of artists to illustrate medical journals and books in the future, and to spare them the years of trial and disappointment of their self-taught predecessors. Practically all medical illustrators are at present self-taught, because there is no school where instruction can be obtained in this branch of art. That some of them have attained a very high degree of proficiency the best class of American text-books very amply prove; and those familiar with the work of Mr. and Mrs. Brödel, the late Mr. Horn, and others of the same school, will agree that no better selection than that of the first-named could have been made for the first professor of this subject.

In announcing the courses proposed for the initial session of his department, Professor Brödel says, quite correctly, that illustrations of medical books are a highly specialised form of art, requiring on the part of the artists not only reliable draughtsmanship and adequate technique, but also a thorough understanding of medicine in most of its branches. It is obvious that a medical illustration can never be a success if the artist does not fully comprehend the object which requires illustration. In other words, the artist ought to have a clear conception of the diagrammatic lesson which his drawing is to convey; and though he must reproduce faithfully the scene which the author puts before him, yet subconsciously he must be influenced by his mental picture of the requirements of the text. The professor announces two courses, one for medical students and a more elaborate one for artists and art students. A careful syllabus is issued of the two courses, from which it is clear that the matter is being dealt with in the thorough and practical spirit that is to be expected both from Mr. Brödel and from the Johns Hopkins University. There is but one comment that we would wish to offer, and that in a very diffident manner. We trust that Mr. Brödel, whose beautiful and artistic drawings are so justly celebrated, will not overlook the value from an educational point of view of line drawings. As an instance of what we mean, we would ask him to compare Dr. Bonney's illustrations in his own and Dr. Berkeley's recent text-book of gynæcological surgery with the elaborate pictures in Dr. Howard Kelly's works (many of which are by Mr. Brödel himself). Labour for labour, Dr. Bonney's are the more economical of the two, and they are certainly almost, if not quite, as effective.

